# Phase Transitions in Bi/Ca Modified AgNbO_3_ Ceramics with Excellent Energy Storage Density and Storage Intensity

**DOI:** 10.1002/smll.202500810

**Published:** 2025-06-16

**Authors:** Zhongna Yan, Jia He, Haiyan Chen, Dou Zhang, Yuan Liu, Hang Luo, Chuanchang Li, Isaac Abrahams, Haixue Yan

**Affiliations:** ^1^ Key Laboratory of Renewable Energy Electric‐Technology of Hunan Province School of Energy and Power Engineering Changsha University of Science and Technology Changsha 410114 China; ^2^ State Key Laboratory of Powder Metallurgy Central South University Changsha 410083 China; ^3^ Department of Chemistry Queen Mary University of London Mile End Road London E1 4NS UK; ^4^ School of Engineering and Materials Science Queen Mary University of London Mile End Road London E1 4NS UK

**Keywords:** AgNbO_3_, antiferroelectric, energy storage, lead‐free, phase transition

## Abstract

Lead‐free antiferroelectric (AFE) ceramics based on AgNbO_3_ represent attractive materials for energy storage applications but are limited by their recoverable energy density (*W*_rec_). Here Bi^3+^/Ca^2+^ A‐site modification of AgNbO_3_ ceramics has yielded a particularly high *W*_rec_ of 4.4 J cm^−3^ and a superhigh recoverable energy storage intensity (*ρ*) of 21.46 × 10^−3^ J kV^−1^ cm^−2^ at 205 kV cm^−1^, the latter being the highest known value obtained at such a relatively low field for a lead‐free ceramic. The modification shifts the dipole freezing temperature, *T*_f_, to below room temperature, enhancing the room temperature stability of the AFE structure. The high *W*_rec_ is attributed to the enhancement of the maximum field‐induced dielectric displacement and improved forward (*E*_F_) and backward (*E*_B_) fields. The work has also allowed for an examination of the poorly understood ±*E*_U_ current peaks evident in current–electric field loops of AgNbO_3_‐based ceramics, which is proposed to be related to a field‐induced AFE to ferroelectric (FE) phase transition in the M_1_ or M_2a_ phases and is absent in the M_2b_ phase due to increased stability of the AFE phase. The exceptional performance of Bi^3+^/Ca^2+^ modified AgNbO_3_ ceramics is promising for potential use in ceramic capacitors for high pulsed power applications.

## Graphical abstract

The debated ±*E*_U_ current peaks are readily distinguished from the ±*E*_2_ current peaks in modified AgNbO_3_ ceramics, which show a particularly high recoverable energy density of 4.4 J cm⁻^3^ and a superhigh energy storage intensity of 21.46 × 10⁻^3^ J kV⁻¹ cm⁻^2^ at a low field of 205 kV cm⁻¹.

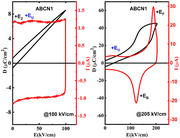

## 1 Introduction

High‐pulsed power technology is widely used in the field of microwave communications, aircraft, hybrid vehicles, high‐pulse power weapons, and laser fusion applications.^[^ ^1^, ^2^, ^3^, ^4^, ^5^ ^]^ Compared with batteries, supercapacitors, and other existing energy storage devices, dielectric capacitors represent the ideal choice for the core components in high‐power pulse applications due to their ultrahigh charge and discharge rates.^[^ ^6^, ^7^ ^]^ At present, commercially available solid‐state capacitors are typically based on polymers and linear dielectric ceramics due to their high energy storage efficiency, but suffer from low energy density, which has prevented their more widespread application.^[^ ^8^, ^9^ ^]^ Increasing the energy density of these systems would benefit important aspects of device design, such as allowing for miniaturization and weight reduction of devices.^[^ ^4^, ^10^ ^]^

For a dielectric material, the recoverable energy density, *W*_rec_, is given by:^[^ ^11^, ^12^ ^]^

(1) $$\;W_{\mathrm{rec}}=\int_{D_{r}}^{D_{m}}E\;dD\;$$

where *D*_m_ is the maximum electrical displacement, *D*_r_ is the remnant electrical displacement and *E* is the applied electric field. Therefore, increasing *D*_m_ and/or *E* and lowering *D*_r_ can lead to higher energy density values. As a result of the increased conduction and hence dielectric loss of polymer‐based capacitors at elevated temperatures, the likelihood of electrical breakdown is high in these devices at higher temperatures and can lead to capacitor failure.^[^ ^13^ ^]^ In contrast, ceramic‐based capacitors have obvious advantages in high‐temperature (>150 °C) applications, such as deep‐well drilling and aerospace industries.^[^ ^14^, ^15^ ^]^ Amongst the four main types of dielectric ceramics, *viz*., linear dielectrics, normal ferroelectrics (FEs), relaxor ferroelectrics (RFEs), and antiferroelectrics (AFEs), the field‐induced double hysteresis loops of AFEs make them particularly good candidates for energy storage applications.^[^ ^2^, ^16^ ^]^

While lead‐containing compounds such as (Pb_0.87_Ba_0.1_La_0.02_)(Zr_0.68_Sn_0.24_Ti_0.08_)O_3_ (PBLZST) have proved commercially successful, serious environmental issues associated with the toxicity of lead and the implementation of relevant policies and regulations in recent years have created a requirement for suitable replacements for lead‐containing ceramics.^[^ ^17^, ^18^, ^19^ ^]^ AgNbO_3_ (AN), a lead‐free AFE ceramic, is considered to be a potential energy storage material due to its double‐like hysteresis loops and large field‐induced polarization of ≈52.0 µC cm^−2^ at 220 kV cm^−1^.^[^ ^20^ ^]^ The discovery of high energy density (2.1 J cm^−3^) in pure AN ceramics ignited research interest in other ceramics based on AN.^[^ ^11^ ^]^ AN exhibits A ferrielectric (FIE) phase at room temperature and contains a large amount of local polar regions, resulting in relatively broad double‐like hysteresis loops, which are not conducive to improving energy storage performance.^[^ ^11^, ^16^, ^20^ ^]^ At present, many studies have mainly focused on shifting the stable AFE phase structure (*i.e*., M_2_ and M_3_ phases) to room temperature through doping methods, and then studying their microstructure and electrical properties. In our previous work, the M_2_ and M_3_ phases of AN ceramics were successfully shifted to room temperature through various doping strategies.^[^ ^6^, ^12^, ^21^ ^]^ A detailed study of the freezing temperature, *T*_f_, in the M_2_ phase was conducted through high‐resolution variable temperature X‐ray powder diffraction (XRD) and Rietveld refinement techniques, showing that *T*_f_ can divide the so‐called M_2_ phase into two phases, namely M_2a_ and M_2b_.^[^ ^21^ ^]^ In addition, local polar regions were observed in the M_2a_, M_2b_, and M_3_ phases through high‐resolution transmission electron microscopy (TEM).^[^ ^6^, ^11^, ^12^, ^21^ ^]^ Based on the extent of these local polar regions distributed in a non‐polar matrix, a basic model for the order of stability of the M‐type AFE phases of AN ceramics was proposed, which increases in the order M_1_ < M_2a_ < M_2b_ < M_3_.^[^ ^21^ ^]^ However, questions still remain regarding AN‐based systems, such as the origin of the weak, but visible, ±*E*_U_ current peaks in current–electric field (*I–E*) loops.

Single‐site doping and co‐doping by cations are effective methods to enhance the energy storage performance of AgNbO_3_ ceramics. For single‐site doping, a significant amount of current research primarily focuses on A‐site (such as Bi^3+^, Ca^2+^, Gd^3+^, La^3+,^ and Sm^3+^) and B‐site (such as Ta^5+^ and W^6+^) doping.^[^ ^16^, ^22^, ^23^, ^24^, ^25^, ^26^, ^27^ ^]^ As for co‐doping, extensive studies have mainly concentrated on A‐site (such as Bi^3+^/Sr^2+^ and Na^+^/La^3+^) and A/B‐site (such as Bi^3+^/Zn^2+^ and Nd^3+^/ Ta^5+^) co‐doping.^[^ ^12^, ^28^, ^29^, ^30^ ^]^ In addition to its notable advantages in phase structure regulation and the enhancement of antiferroelectricity, co‐doping also allows for the flexible selection of corresponding cations based on the characteristics of single‐site doping. The synergistic effects of multiple cations can be used to strengthen *D*_m_ and breakdown strength. For instance, AgNbO_3_ ceramics co‐doped with Bi^3+^/Sr^2+^ and Na^+^/La^3+^ on the A‐site have achieved super‐high energy storage densities of 7.9 and 11.4 J cm^−3^ at fields of 702 and 670 kV cm^−1^, respectively.^[^ ^28^, ^29^ ^]^ Similarly, high energy densities of 4.6 and 6.5 J cm^−3^ at fields of 220 and 370 kV cm^−1^ were obtained, respectively, in Bi^3+^/Zn^2+^ and Nd^3+^/ Ta^5+^ co‐doped AgNbO_3_ ceramics.^[^ ^12^, ^30^ ^]^ Tian et al. reported that doping Bi^3+^ onto the A‐site can lower the *T*_f_ to low temperatures and shift the transition peaks associated with the forward (*E*_F_) and backward (*E*_B_) electric fields to higher values. Bi^3+^ doping can also decrease the difference between *E*_F_ and *E*_B_, which is related to higher energy storage efficiency.^[^ ^25^ ^]^ Luo et al. reported that doping of Ca^2+^ onto the A‐site can not only generate similar effects to those of the Bi^3+^ cation, but also improve the field‐induced *D*_m_ through the introduction of A‐site vacancies required to maintain electroneutrality.^[^ ^26^ ^]^ Ca^2+^ doping can also decrease grain size, leading to an increased breakdown field, which is useful for increasing energy density. In addition, it has been reported that *D*_m_ can be further increased through hybridization between the O 2p and Bi 6s orbitals in a similar way to that reported to occur in Pb‐based systems.^[^ ^18^, ^31^ ^]^

In the present study, in order to combine the advantages of Bi^3+^ doping relating to improved efficiency and Ca^2+^ doping relating to higher breakdown fields, Bi^3+^ and Ca^2+^ co‐doping on the A‐site was used to further increase the energy storage density and efficiency of AN‐based systems. In addition, based on the shift of *T*_f_ we attempt to recognize the nature of the weak ±*E*_U_ current peaks. A particularly high recoverable energy density of ≈4.4 J cm^−3^ and a superhigh recoverable energy storage intensity of ≈21.46 × 10^−3^ J kV^−1^ cm^−2^ have been obtained at a relatively low applied field of 205 kV cm^−1^. This recoverable energy storage intensity ranks as one of the highest known values obtained at such a relatively low field in a lead‐free ceramic system.

## 2 Results and Discussion

**Figure 1:**
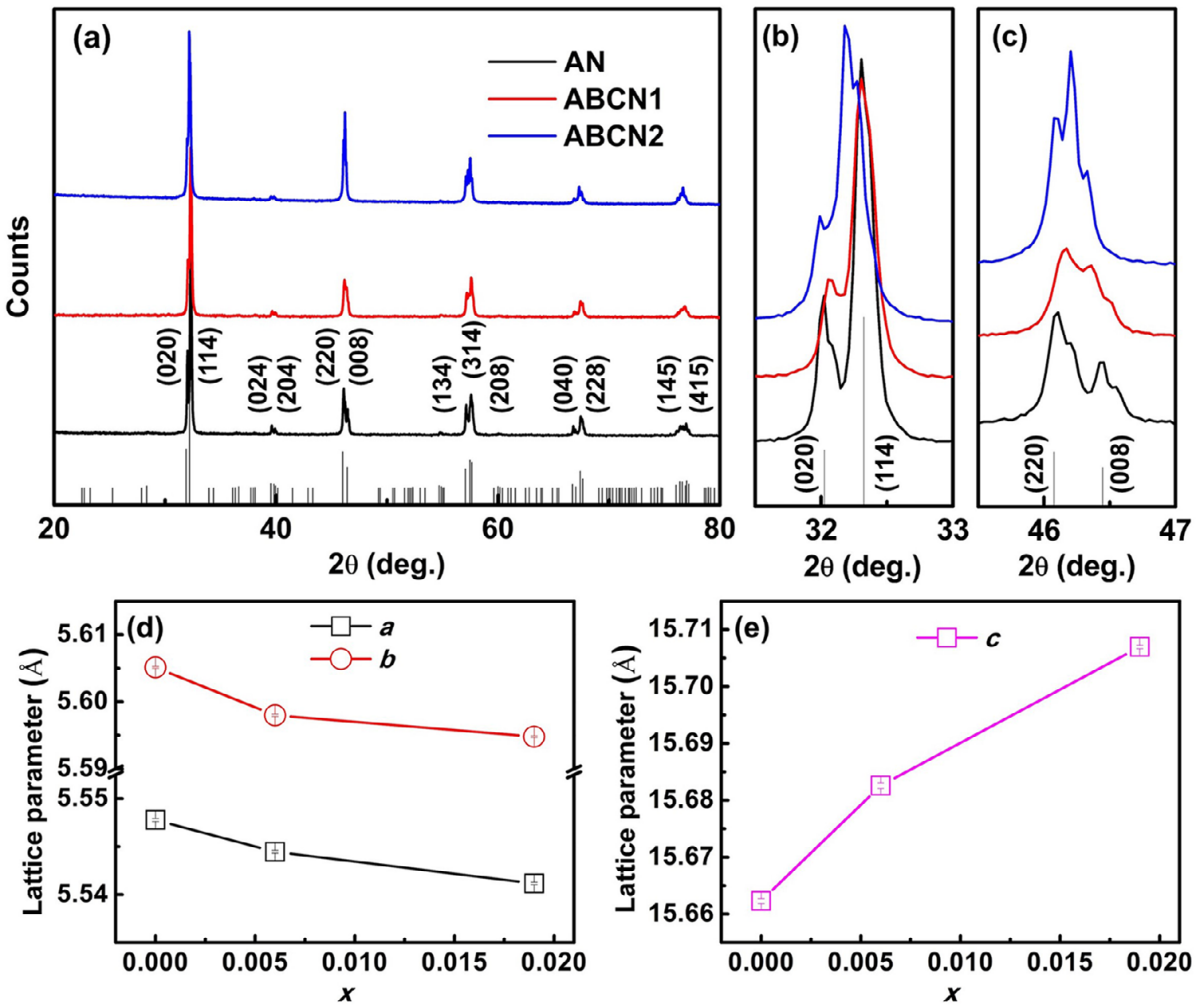
a) XRD patterns of AN, ABCN1, and ABCN2 ceramic powders with detail shown in (b,c); d,e) compositional variation of refined lattice parameters (error bars are shown in the open symbols).

The XRD patterns of the studied ceramic powders (**Figure** **1**a) are similar to each other, and that for the *x* = 0.000 composition matches the standard pattern for AN (PDF # 70–4738). Close inspection of the peaks at ≈32° and 46° 2θ (Figure 1b,c) reveals some subtle changes compared to the pattern of pure AN. The (114) and (200) peaks at ≈32.3° 2θ, which completely overlap in pure AN, become more separated with increasing *x*‐value, while the separation between these peaks and that for (020), at ≈32.0° 2θ, reduces. Similarly, the (008), (221), (125), (215) and (117) reflections appear as a single broad peak in pure AN at ≈46.5° 2θ, which is well separated from the (220) peak at ≈46.1° 2θ, but for the *x* = 0.006 and *x* = 0.019 compositions this separation is diminished.

Figure 1d,e shows the compositional variation of lattice parameters in the AN, ABCN1, and ABCN2 ceramics derived from Rietveld fits to the XRD profiles (Figure S1, Supporting Information). The *a* and *b* cell parameters decrease with increasing *x*‐value, as the average radius of the A site cation decreases, as expected for the replacement of Ag^+^ by Bi^3+^ and Ca^2+^, with ionic radii of 1.28, 1.17, and 1.12 Å, respectively, in eight coordinate geometry.^[^ ^32^ ^]^ In contrast, the *c*‐axis shows an increasing trend with increasing *x*‐value. It should be noted here that an increase in the *c*‐parameter and small decreases in the *a* and *b* cell parameters have been reported for the thermally induced M_1_ to M_2_ transition in Li‐doped AgNbO_3_,^[^ ^33^ ^]^ as the average structure changes symmetry from polar *Pb*2_1_ *m* to non‐polar *Pbcm*. As we discuss below, in the present case this transition is compositionally induced between *x* = 0.000 and *x* = 0.019.

To explain the overall trends in lattice parameters, it is helpful to consider the structural effects of Bi^3+^/Ca^2+^ incorporation into the AN system. AN shows an orthorhombically distorted perovskite structure, with (*a* ^−^ *,b* ^−^ *,c^+^*)/(*a* ^−^ *,b* ^−^ *,c* ^−^) tilting of the B‐site octahedra. As the *a* and *b* axes shrink in size with Bi^3+^/Ca^2+^ incorporation, the tilt angle with respect to the *c*‐direction would be expected to decrease, leading to a lengthening of the *c*‐axis, as observed. The orthorhombic lattice parameters (o) are related to those of the perovskite subcell (p) by *a*_o_ = √2*a*_p_, *b*_o_ = √2*a*_p_ and *c*_o_ = 4*a_p_*. The standard deviation in the perovskite subcell dimensions decreases with increasing *x*‐value, suggesting the structure approaches a more cubic‐like geometry.

**Figure 2:**
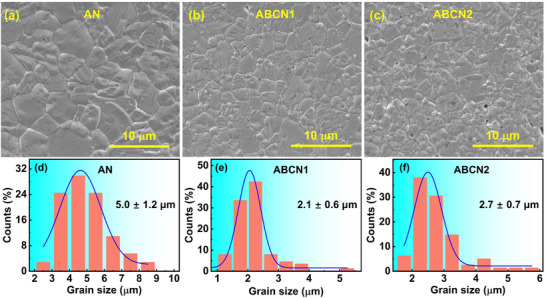
a–c) SEM images and d–f) variation in the grain sizes of AN, ABCN1, and ABCN2 ceramics. Data were collected from 100 randomly distributed grains.

**Figure** **2**a–c shows the SEM images of AN, ABCN1, and ABCN2 ceramics. All ceramics have dense microstructures with little porosity, and relative densities higher than 95%. There is significant variation in the grain sizes in all compositions with the average grain size ranging from 2.1 µm for the *x* = 0.006 composition to 5.0 µm in pure AN. A uniform distribution of elemental components is shown in EDS mapping images of the ABCN1 ceramics (Figure S2, Supporting Information).

**Figure 3:**
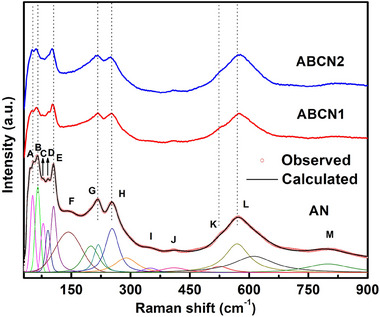
Raman spectra of AN, ABCN1, and ABCN2 ceramic powders.

**Figure** **3** shows the Raman spectra of the studied AN, ABCN1, and ABCN2 ceramic powders. The spectrum for AN matches well with that was previously reported.^[^ ^34^ ^]^ The peaks A and B, at low wavenumber, are ascribed to the shifts of Ag^+^/Nb^5+^cations, peak C is ascribed to the shift of Ag^+^ cations, while peak D is ascribed to the NbO_6_ tilting.^[^ ^34^ ^]^ The peaks G, J, and K, at high wavenumber, are ascribed to the *υ*_5_ (triply degenerate symmetric bending) *υ*_2_, and *υ*_1_ (doubly degenerate symmetric stretching) internal modes of NbO_6_ octahedra.^[^ ^16^, ^27^, ^34^ ^]^ Compared with pure AN, peaks A, B, C, and J become less distinct, while peaks D, J, and K become broader and shift, indicating greater disorder after incorporation of Bi^3+^ and Ca^2+^.

**Figure 4:**
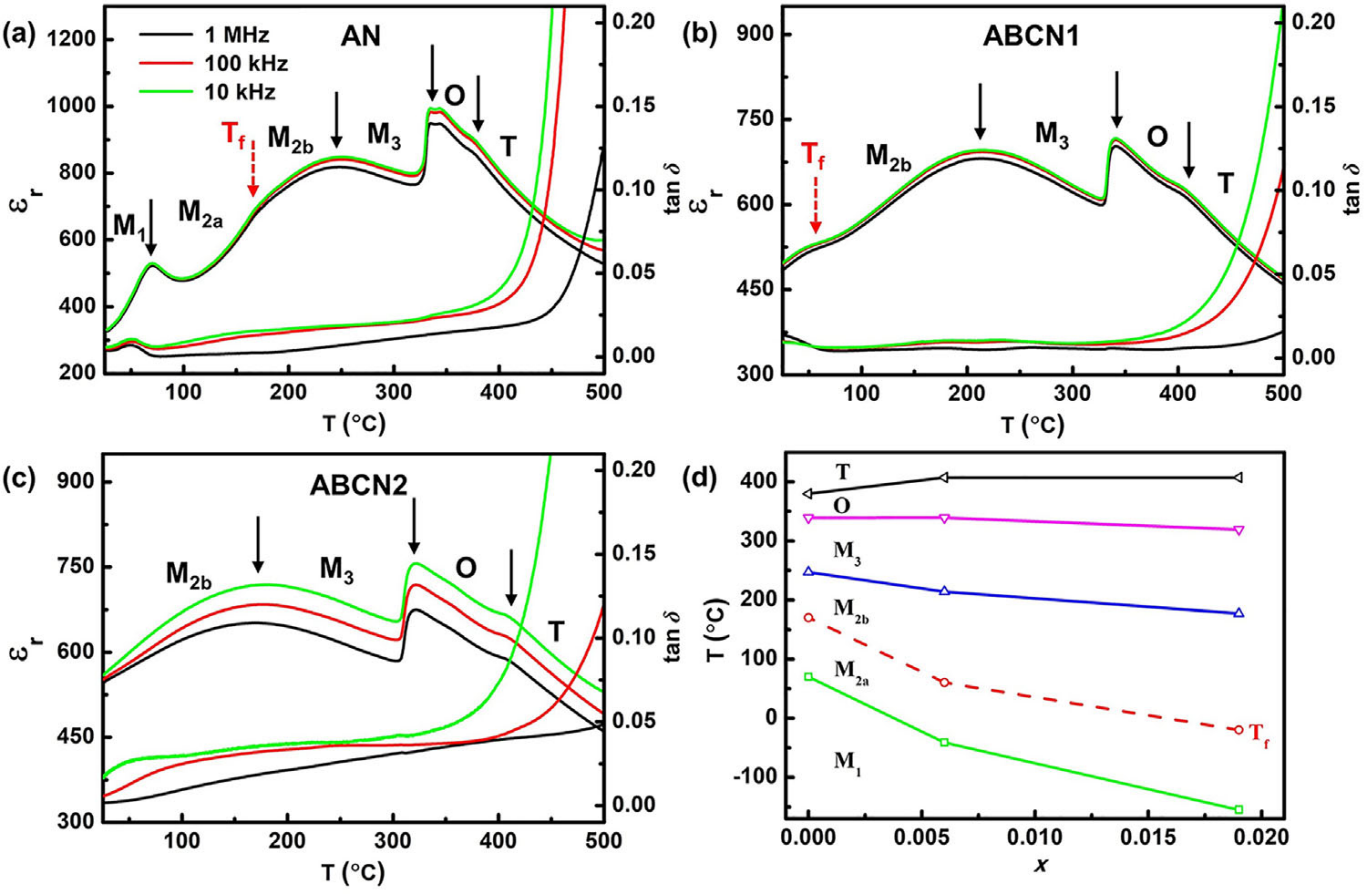
a–c) Temperature dependencies of the relative permittivity (ε_r_) and loss tangent (tan *δ*) of AN, ABCN1, and ABCN2 ceramics from 10 kHz to 1 MHz; d) phase diagram for the Ag_(1‐5_ *_x_*_)_Bi*_x_* Ca*_x_* NbO_3_ system.

The relative permittivity and dielectric loss on heating AN, ABCN1, and ABCN2 ceramics from ambient temperature to 500 °C in the frequency range 10 kHz–1 MHz are shown in **Figure** **4**a–c. Sub‐ambient temperature data are given in Figure S3 (Supporting Information). The data for AN shown in Figure 4a reveals a number of dielectric anomalies, corresponding to the previously reported phase transitions in AN.^[^ ^11^, ^20^ ^]^

A weak dielectric anomaly at ≈170 °C has previously been ascribed to the dipole freezing temperature, *T*_f_.^[^ ^35^ ^]^ It has been proposed that *T*_f_ serves as the Curie point of the ferrielectric phase in AN ceramics.^[^ ^36^ ^]^ We have recently proposed that since *T*_f_ lies inside the existence range of the so called “M_2_ phase”, then this region needs to be considered as covering two phases, with the phase below *T*_f_ denoted as M_2a_ and the phase above *T*_f_ denoted as M_2b_.^[^ ^21^ ^]^ Therefore, there are four M phases, namely M_1_, M_2a_, M_2b_, and M_3_, which are reported to possess orthorhombic symmetry^[^ ^11^, ^37^ ^]^ (the “M” designation arises from the fact they were originally thought to be monoclinic).^[^ ^38^ ^]^ The M_1_ → M_2a_, M_2a_ → M_2b_, and M_2b_ → M_3_ phase transitions occur at ≈70, 170, and 250 °C, respectively. The O and T high‐temperature paraelectric phases have orthorhombic and tetragonal symmetries, respectively, with well‐characterized structures in their respective space groups of *Cmcm* and *P*4/*mbm*.^[^ ^39^ ^]^ The phase transitions, M_3_ → O and O → T, are seen to occur at ≈330 and 380 °C, respectively. Similar features are observed in the plots for the ABCN1 and ABCN2 ceramics, but with increasing *x*‐value, the existence ranges of the phases increasingly shift to lower temperatures. As a consequence of this, while in AN M_1_ is the stable room temperature phase, the stability range of the M_1_ phase shifts to sub‐ambient temperature in the ABCN1 ceramic (Figure S3, Supporting Information), leaving the M_2a_ phase present at room temperature. Similarly, in the ABCN2 ceramic, both the M_1_ ↔ M_2a_ phase transition and *T*_f_ shift to sub‐ambient temperatures, leaving M_2b_ as the room temperature stable phase. The various phase transitions of AN, ABCN1, and ABCN2 ceramics are summarized in the phase diagram shown in Figure 4d.

**Figure 5:**
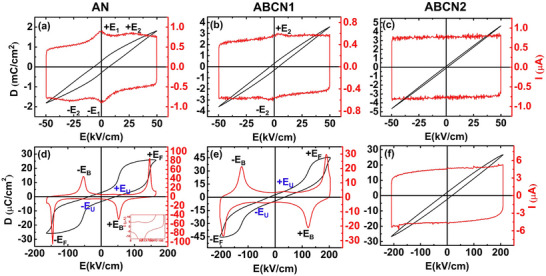
Ferroelectric *D–E* and *I–E* loops measured at 1 Hz for a,d) AN, b,e) ABCN1, and c,f) ABCN2 ceramics at (a–c) low and (d–f) high applied fields; the inset in (d) shows detail of the +*E*_U_ peak.

*D–E* and *I–E* loops at low‐fields (50 kV cm^−1^) and high‐fields (165, 205, and 210 kV cm^−1^ for AN, ABCN1, and ABCN2, respectively) measured at 1 Hz are shown in **Figure** **5**. Two significant current peaks, viz., ±*E*_1_ ≈ ±1 kV cm^−1^ and ±*E*_2_ ≈ ±27 kV cm^−1^, are seen in the *I–E* loop of the AN ceramic (Figure 5a in red color). ±*E*_1_ is ascribed to the switching of local domains in the M_1_ phase, while ±*E*_2_ corresponds to a field‐induced transition to an FIE phase, the Curie point of which lies at *T*_f_.^[^ ^36^ ^]^ After modification, the ±*E*_1_ current peak, which is related to the M_1_ phase, is not observed at a low field in both ABCN1 and ABCN2 ceramics (Figure 5b,c), as the permittivity data confirm these two compositions to be in the M_2a_ and M_2b_ phases, respectively (Figure 4b,c). The ±*E*_2_ peak is observed in the ABCN1 ceramic and becomes broader (Figure 5b) but is absent in the *I–E* loop of the ABCN2 ceramic (Figure 5c). This is consistent with the shifting of the M_1_ → M_2a_ and *T*_f_ transitions to sub‐ambient temperature in the ABCN2 ceramic. A characteristic double‐like *D–E* loop with two significant current peaks, *E*_F_ (forward electric field, corresponding to an AFE → FE transition) and *E*_B_ (backward electric field, corresponding to the reverse FE → AFE transition) in the *I–E* loop are seen in the pure AN ceramic (Figure 5d), indicating a reversible field induced phase transition, consistent with previous reports.^[^ ^11^, ^20^ ^]^ After modification, a similar *D–E* hysteresis loop and two strong current peaks, *E*_F_ and *E*_B_, can be observed in the ABCN1 ceramic (Figure 5e). However, a dramatic change occurs for the ABCN2 ceramic, with no current peaks in the *I–E* loop and a near linear *D–E* loop, which broadens somewhat at higher fields. This indicates a pure capacitor contribution at low fields and an additional loss contribution at high fields (Figure 5f). These results indicate that the ABCN2 ceramic is not an AFE phase or that the field‐induced AFE to FE transition in the M_2b_ phase does not readily occur at these applied fields. A weak current peak, ±*E*_U_, can be observed through careful inspection of the *I–E* loop in the AN ceramic (Figure 5d), consistent with previous reports.^[^ ^11^, ^12^ ^]^ A similar ±*E*_U_ peak can also be observed in the ABCN1 ceramic but is absent in the ABCN2 ceramic.

**Figure 6:**
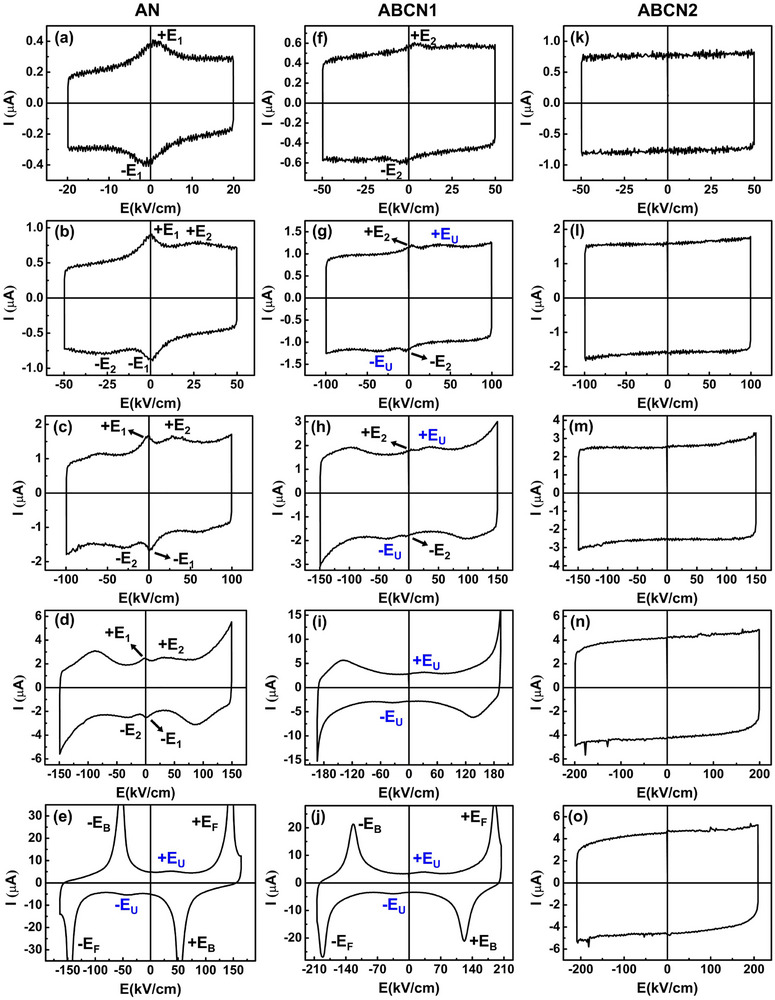
*I–E* loops for a–e) AN, f–j) ABCN1, and k–o) ABCN2 ceramics measured at different electric fields at 1 Hz.

**Figure** **6** shows the *I–E* loops of AN, ABCN1, and ABCN2 ceramics at different applied electric fields. As shown in Figure 6b–d, there are two current peaks, ±*E*_1_ and ±*E*_2_, for AgNbO_3_ ceramics. The ±*E*_U_ peak in Figure 6e is a current peak that appears at low fields when AgNbO_3_ shows AFE behavior under high applied electric fields. The field of the ±*E*_U_ current peak is very close to that of the ±*E*_2_ peak, and so these peaks are not easily distinguishable. In contrast, at 50 kV cm^−1^ the ABCN1 ceramic shows the ±*E*_2_ peak, with the ±*E*_U_ peak becoming more visible only at higher fields of 100 kV cm^−1^ and above (Figure 6h–j). In the case of the ABCN2 ceramic, the *D–E* loop changes little with increasing applied field, and no current peaks are visible at fields up to 210 kV cm^−1^ (Figure 6k–o). This shows that there are two transitions in AgNbO_3_‐based materials under high applied fields. The main transition shows a reversible field‐induced structure change from AFE to FE, which has high polarization. The other transition is an irreversible field‐induced transition, which is related to the ±*E*_U_ peak at low field, and can be induced in the M_1_ and M_2a_ phases at high fields, but not in the M_2b_ phase in the field ranges applied.

**Figure 7:**
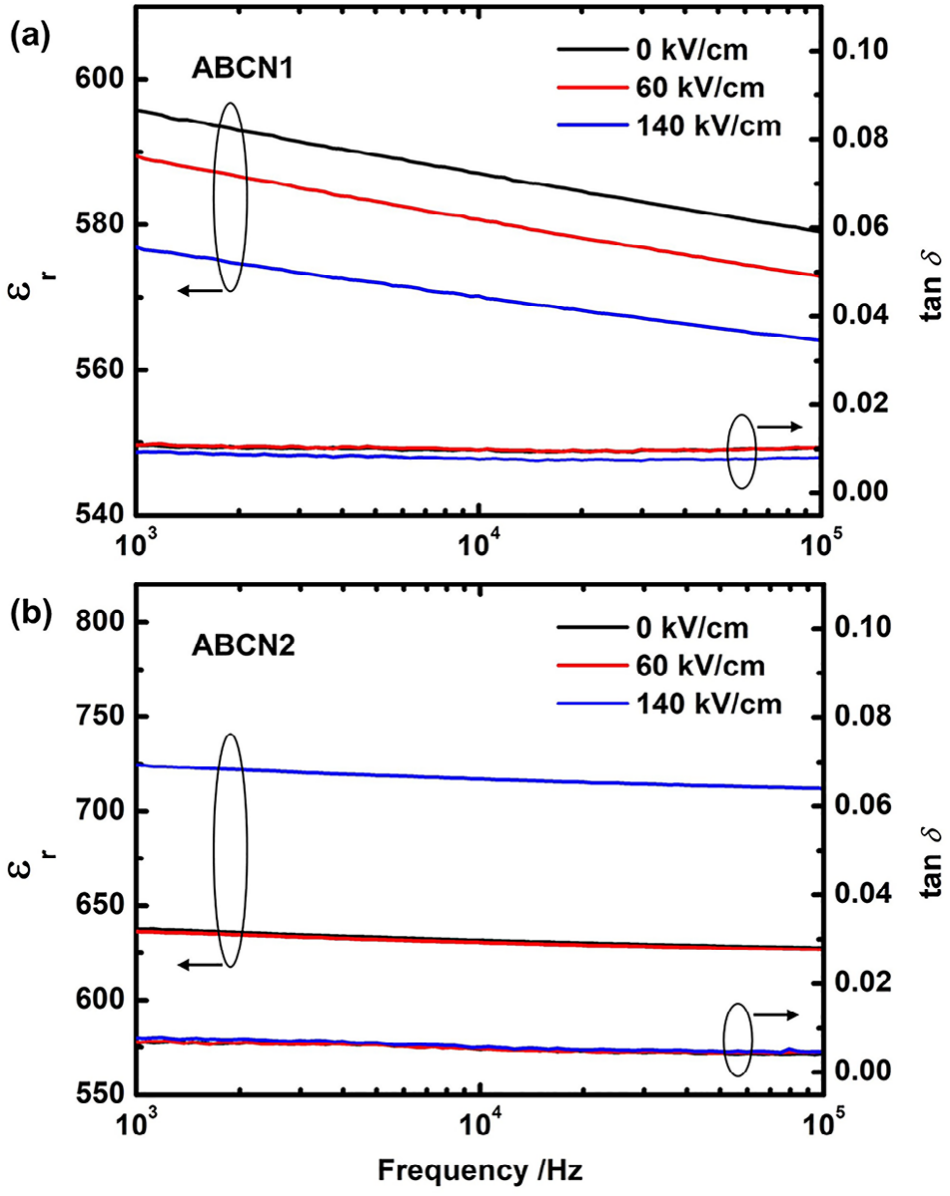
Room temperature frequency dependencies of the relative permittivity (ε_r_) and loss tangent (tan *δ*) in AN, ABCN1, and ABCN2 ceramics prior to and post DC poling for 5 min at 60 and 140 kV cm^−1^.

Dielectric permittivity and loss, as functions of frequency, before and after DC poling for 5 min, are shown in **Figure** **7**. After DC poling at 60 or 140 kV cm^−1^, there is a continuous decrease in dielectric permittivity of the ABCN1 ceramic, which is primarily associated with switching of the local polar regions in the AFE matrix, giving rise to a continuous reduction in domain wall density.^[^ ^12^, ^21^, ^40^, ^41^ ^]^ These local polar regions have previously been directly observed and give rise to additional reflections in electron diffraction patterns.^[^ ^11^ ^]^

For the ABCN2 ceramic, there is only a small observable decrease in dielectric permittivity, and this is mainly a result of the reduction of domain wall density, possibly associated with low concentrations of residual local polar regions. In addition, an increase in dielectric permittivity, seen in the ABCN2 ceramic poled at 140 kV cm^−1^, suggests the creation of new polar regions, which indicates that a phase transition from AFE to FE might occur at higher applied fields.^[^ ^12^, ^21^ ^]^ However, as seen in Figure 5f, this transition, if accessible considering possible electrical breakdown, lies above the maximum field of 1 Hz used in the present study.

**Table** **1** summarizes the energy storage performances of AN, ABCN1, and ABCN2 ceramics measured at 1 Hz. *D*_m_, increases from 26.0 µC cm^−2^ for pure AN to 45.1 µC cm^−2^ for the ABCN1 ceramic, and then decreases to 26.7 µC cm^−2^ for the ABCN2 ceramic. The high *D*_m_ suggests that the Bi^3+^/Ca^2+^ co‐doping and incorporation of A‐site vacancies can increase the field‐induced polarization in doped AgNbO_3_ materials. The same trend is observed in *D*_r_, which slightly increases from 2.7 µC cm^−2^ for pure AN to 2.8 µC cm^−2^ for ABCN1 and decreases to 2.2 µC cm^−2^ for the ABCN2 ceramic (Figure 5d–f, Table 1). Compared with the pure AN ceramic, after modification, peaks *E*_F_ and *E*_B_, corresponding to electric field‐induced transitions, are shifted to higher electric fields in the ABCN1 ceramic, consistent with the inhibition of FE ordering in the local polar regions, which favors the stability of the AFE structure.^[^ ^16^, ^23^, ^26^, ^27^ ^]^ The large value of *D*_m_ (45.1 µC cm^−2^), along with the increased *E*_F_ and *E*_B_ values, results in a maximum *W*_rec_ of 4.4 J cm^−3^ in the ABCN1 ceramic (Figure 5e). If a higher field could be applied to ABCN2, it is possible that better energy storage performance might be achieved. The breakdown field of ABCN2 is possibly related to its large grain size (Figure 2), and high dielectric permittivity (Figure 7).

**Table 1:** Energy storage performances of AN, ABCN1, and ABCN2 ceramics.

Samples	*D* [µC cm^−2^]		*E* [kV cm^−1^]				*W*_rec_ [J cm^−3^]	*η* [%]	*ρ*
	*D*_m_	*D*_r_	*E*_F_	*E*_B_	Δ*E*_F‐B_	*E*_b_			[×10^−3^ J kV^−1^ cm^−2^]
AN	26.0	2.7	144.9	52.1	92.8	165	1.3	37.7	7.88
ABCN1	45.1	2.8	189.3	122.6	66.7	205	4.4	63.0	21.46
ABCN2	26.7	2.2	–	–	–	210	2.4	77.5	11.43

**Figure 8:**
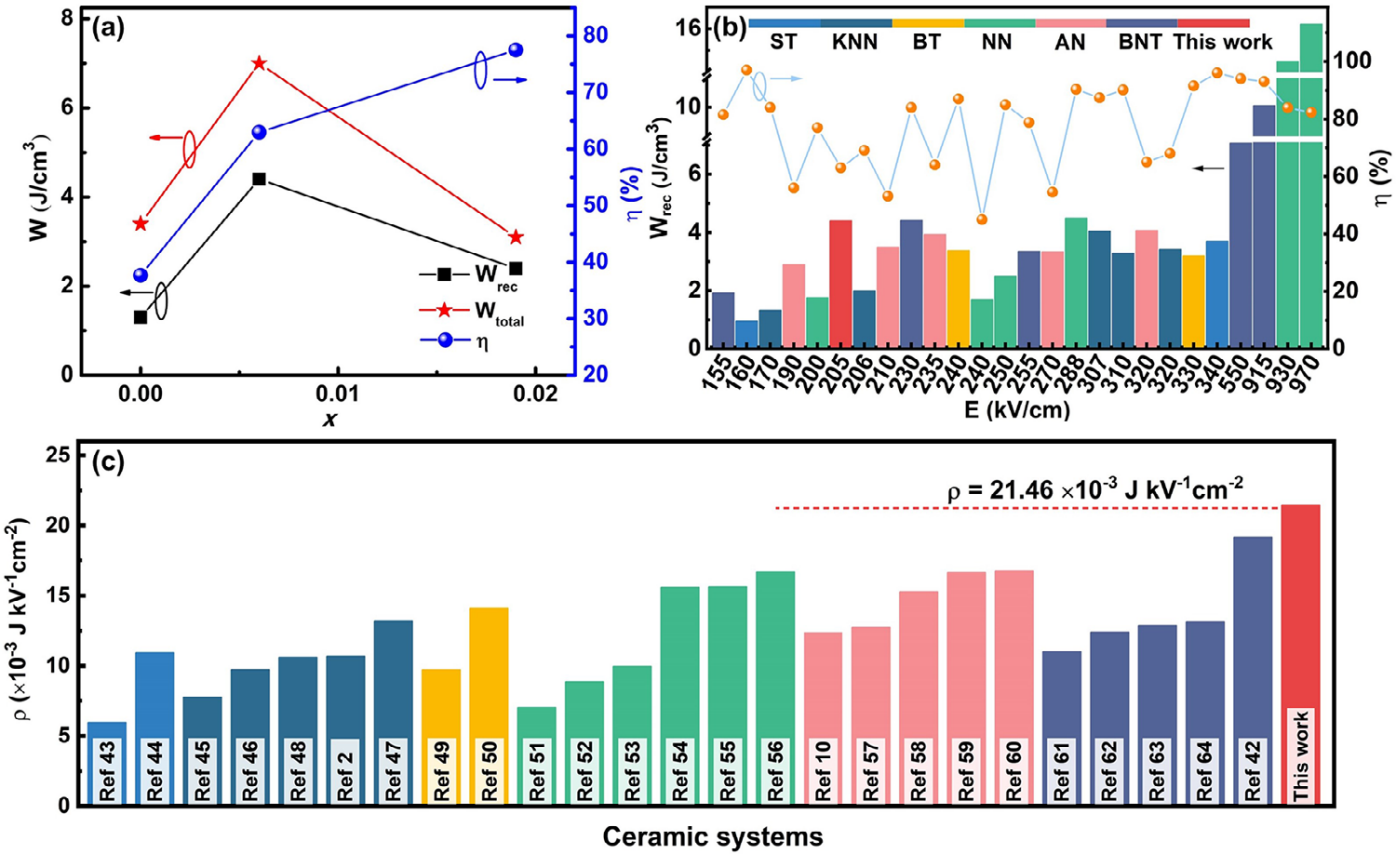
a) Energy storage properties of AN, ABCN1, and ABCN2 ceramics; b) recoverable energy storage density and energy storage efficiency, and c) recoverable energy storage intensity for ABCN1 ceramic compared to that in other recently reported lead‐free ceramic systems.

Energy storage efficiency, *η*, is given by:^[^ ^16^, ^25^ ^]^

(2) $$\eta =\frac{W_{\mathrm{rec}}}{W_{\mathrm{rec}}+W_{\mathrm{loss}}}\;$$

where *W*_rec_ is the recoverable energy density and *W*_loss_ is the energy density loss. *η* values are seen to show an increasing trend with increasing levels of substitution (**Figure** **8**a). Compared to pure AN, the high *η* of 63.0% in ABCN1 is mainly due to the difference (Δ*E*_F‐B_) between *E*_F_ and *E*_B_, decreasing from 92.8 kV cm^−1^ for the AN ceramic to 66.7 kV cm^−1^ for the ABCN1 ceramic. Although the electric field‐induced phase transition peaks, *E*_F_ and *E*_B_, were not observed in the ABCN2 ceramic, the enhanced stability of the AFE structure in the M_2b_ phase is the main reason for the high efficiency of 77.5%. We have recently proposed that recoverable energy storage intensity, *ρ*, can be used to assess energy storage materials based on the energy density determined under a certain electric field. The recoverable energy storage intensity (*ρ*) is given by:^[^ ^42^ ^]^

(3) $$\rho =\frac{W_{\mathrm{rec}}}{\Delta E}\;$$

(4) $$\Delta E=E_{b}\;-\;E_{s}$$

where *E*_b_ is the breakdown electric field and *E*_s_ is the starting electric field (normally defined as 0 kV cm^−1^). The calculated room‐temperature values of *ρ* for AN, ABCN1, and ABCN2, according to Equations (3) and (4), are summarized in Table 1. The ABCN1 sample shows the highest *ρ* value of ≈21.46 × 10^−3^ J kV^−1^ cm^−2^ at a field of 205 kV cm^−1^ at room temperature. This indicates that high energy density can be achieved in the material under low voltage, which is very important for practical applications such as high‐power energy storage devices because the devices normally operate under applied fields of less than half of the dielectric breakdown strength for safety reasons. Moreover, this composition achieves a particularly high recoverable energy storage density (Figure 8b) and a maximum recoverable energy storage intensity (Figure 8c) at an electric field that is lower than those reported for almost any other lead‐free ceramic system, including K_0.5_Na_0.5_NbO_3_ (KNN), BaTiO_3_ (BT), Bi_0.5_Na_0.5_TiO_3_ (BNT), SrTiO_3_ (ST), and NaNbO_3_ (NN) based systems, as well as other AN based ceramics.^[^ ^2^, ^10^, ^42^, ^43^, ^44^, ^45^, ^46^, ^47^, ^48^, ^49^, ^50^, ^51^, ^52^, ^53^, ^54^, ^55^, ^56^, ^57^, ^58^, ^59^, ^60^, ^61^, ^62^, ^63^, ^64^ ^]^

**Figure 9:**
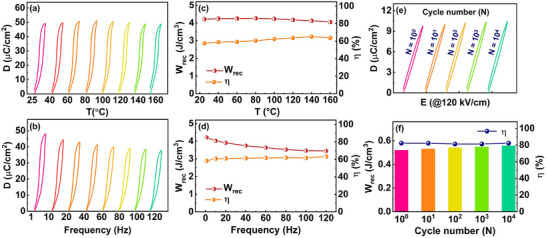
a,c) Temperature and b,d) frequency stabilities, as well as e,f) cycling stability of energy storage properties of the ABCN1 ceramic.

The temperature and frequency stability of energy storage properties are important issues for dielectric ceramics in practical applications. **Figure** **9**a shows the temperature‐dependent unipolar *D–E* loops of the ABCN1 ceramic measured at 200 kV cm^−1^ with a frequency of 1 Hz. As temperature increases, *D*_m_ slightly rises, reaching its maximum value at 80 °C. Subsequently, as the temperature rises to 160 °C, *D*_m_ gradually decreases to a level nearly identical to that at room temperature. The main reason for the local increase in *D*_m_ value is likely related to the M_2a_‐M_2b_ phase transition occurring at ≈80 °C (*i.e*., the *T*_f_ temperature), as shown in Figure 4b. Moreover, as the temperature increases, the *D–E* loops become increasingly narrower. As a result, both the *W*_rec_ and *η* values are thermally stable up to 160 °C (Figure 9c), indicating that the ABCN1 ceramic exhibits excellent thermal stability in energy storage over a wide temperature range.

Figure 9b shows the frequency dependence of unipolar *D–E* loops of the ABCN1 ceramic measured at 200 kV cm^−1^ over the frequency range of 1–120 Hz. It can be observed that as the frequency increases, the *D*_m_ value of the ABCN1 ceramic gradually decreases, while the *D–E* loops become increasingly narrow, resulting in a slight decrease of *W*_rec_ and a slight increase of *η* up to 120 Hz (Figure 9d). This indicates that the ABCN1 ceramic exhibits good frequency stability. In addition, the cycling stability of the ABCN1 ceramic was measured at 120 kV cm^−1^ (nearly half of the breakdown strength, adopted to ensure safe operation,)^[^ ^65^, ^66^ ^]^ at a frequency of 10 Hz (Figure 9e,f). The unipolar *D–E* loops retain a narrow profile after 10^5^ cycles, with minimal changes in *D*_m_, *W*_rec_, and *η*, thus demonstrating excellent cycling stability at room temperature.

**Figure 10:**
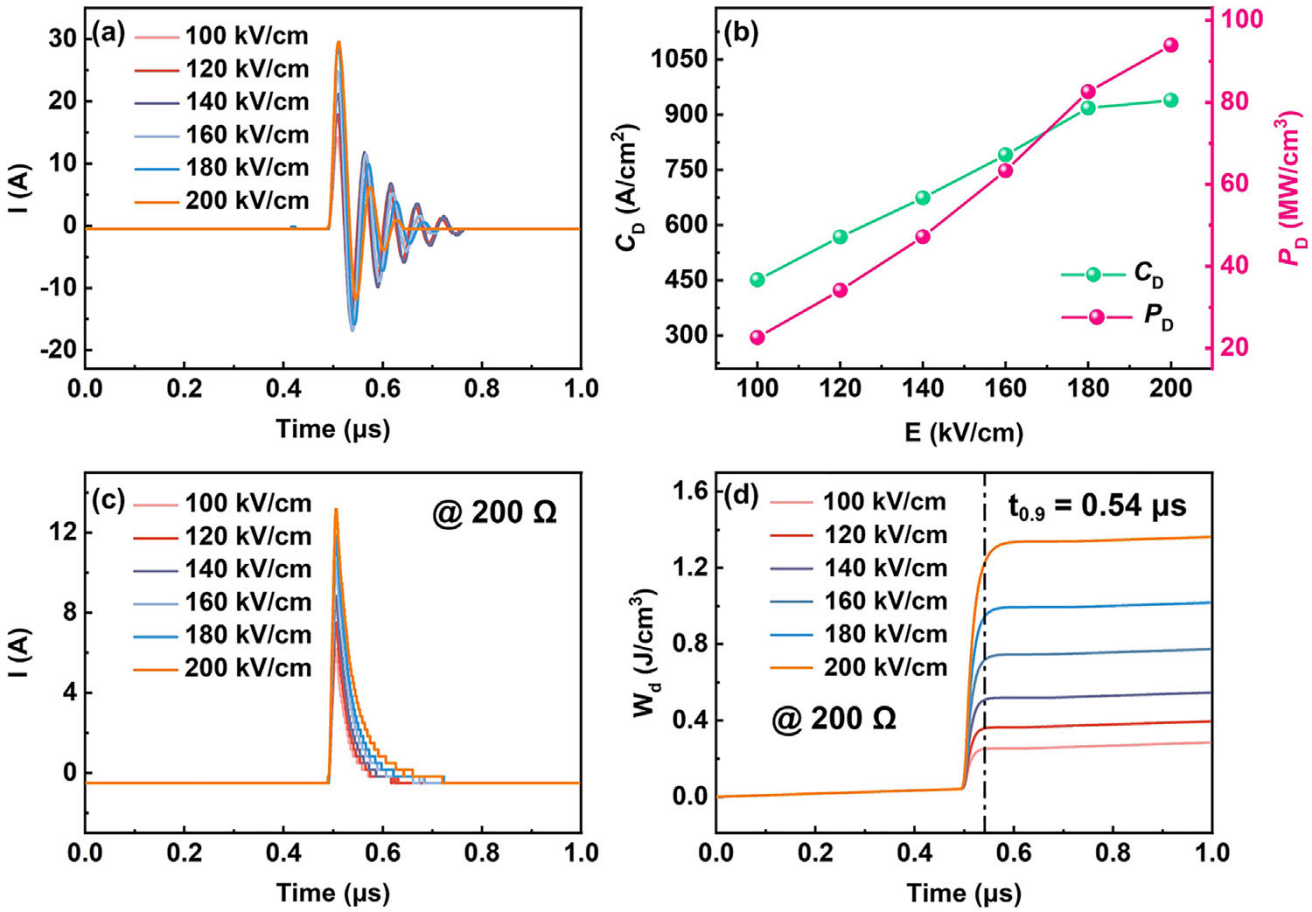
The charge–discharge performance of the ABCN1 ceramic.

The charge–discharge characteristics of dielectric capacitors are also crucial in the evaluation of their actual performance. **Figure** **10**a shows the underdamped discharge current waveforms of the ABCN1 ceramic under different electric fields. All the current waveforms present a similar discharge behavior, and the current peak (*I*_max_) increases with increasing electric field. The values of current density (*C*_D_) and power density (*P*_D_) can be calculated as follows:^[^ ^53^, ^55^ ^]^

(5) $$C_{D}=\frac{I_{max}}{S}$$

(6) $$P_{D}=\frac{E\times I_{max}}{2S}$$

where *S* is the electrode area of the samples and *E* is the electric field. Figure 10b shows the *C*_D_ and *P*_D_ values of the ABCN1 ceramic under various electric fields. *C*_D_ and *P*_D_ gradually increase with increasing electric field, with maximum values of 939.49 A cm^−2^ and 93.94 MW cm^−3^ achieved at 200 kV cm^−1^, respectively, illustrating promising potential in high pulsed power technology.

Figure 10c,d shows the overdamped discharge current waveforms and calculated discharge energy density (*W*_d_) of the ABCN1 ceramic under different electric fields. *W*_d_ can be calculated using Equation (7):^[^ ^54^, ^56^ ^]^

(7) $$W_{d}=\frac{R\int I^{2}\left(t\right)dt}{V}\;$$

where *R*, *I*, *t*, and *V* are the load resistance (200 Ω in this study), discharge current, time, and sample volume (5.966 × 10^−4^ cm^3^ in this study), respectively. As shown in Figure 10d, *W*_d_ increases with increasing electric field, with a maximum value of 1.36 J cm^−3^ achieved at 200 kV cm^−1^, which is smaller than the value of *W*_rec_ calculated from the *D–E* loops at a similar electric field. This difference is likely to be primarily caused by the duration of the applied electric field utilized in both methods. The DC charge–discharge process is typically completed in <1 ms, which would lead to clamping and incomplete switching of AFE domains.^[^ ^56^, ^66^ ^]^ To assess the charge–discharge rate, the time corresponding to 90% of discharged energy is defined as charge–discharge time (*t*_0.9_). A *t*_0.9_ of ≈0.54 µs is obtained in the ABCN1 ceramic at 200 kV cm^−1^, as displayed in Figure 10d.

## 3 Conclusions

In this work, a particularly high *W*_rec_ of 4.4 J cm^−3^ and a super‐high *ρ* of 21.46 × 10^−3^ J kV^−1^ cm^−2^ were obtained in a Bi^3+^/Ca^2+^ modified AN ceramic at a low electric field of 205 kV cm^−1^. A shifting of *T*_f_ to below ambient temperature occurs as a result of A‐site Bi^3+^/Ca^2+^ incorporation, which is the main reason for the enhancement of the stability of the AFE structure. The ±*E*_U_ current peaks, which have up to now remained unexplained are readily distinguished from the ±*E*_2_ current peaks after Bi^3+^/Ca^2+^ modification and appear to be associated with an AFE → FE field‐induced phase transition. The enhancement of *D*_m_ combined with the increased *E*_F_ and *E*_B_ electric fields, result in a high *W*_rec_. In addition, good thermal, frequency, and cycling stabilities were achieved in ABCN1 ceramics, which also displayed a large *C*_D_ of 939.49 A cm^−2^ and *P*_D_ of 93.94 MW cm^−3^ at 200 kV cm^−1^. These features make the ABCN1 ceramic a promising candidate material for use in high‐performance ceramic capacitors in energy storage applications.

## 4 Experimental Section

Ceramic samples of AN (AgNbO_3_), ABCN1 (Ag_(1−5_ *_x_*_)_Bi*_x_* Ca*_x_* NbO_3_, where *x* = 0.006), ABCN2 (Ag_(1−5_ *_x_*_)_Bi*_x_* Ca*_x_* NbO_3_, where *x* = 0.019) were prepared through the solid‐state reaction of the precursor oxides. Additional Bi_2_O_3_ and Ag_2_O (5*x*/2 Ag_2_O + *x*/2 Bi_2_O_3_) were added to the powders to compensate for the volatilization of these oxides during synthesis. Ag_2_O (≥99.7%), Bi_2_O_3_ (≥99.9%), Nb_2_O_5_ (≥99.99%), and CaCO_3_ (≥98%) were milled in ethanol in a nylon jar using a planetary ball mill for 24 h at 250 rpm. The dried mixtures were calcined at 880 °C in alumina crucibles for 6 h in flowing O_2_ (0.5 L min^−1^). After cooling, the powders were milled in ethanol for a further 24 h and then dried at 200 °C. Pellets of 10 mm diameter and 1–2 mm thickness were pressed from the dried powders at a pressure of 180 MPa for 1 min and then sintered between 1075–1090 °C for 6 h in flowing O_2_ (1090 °C for AN, 1080 °C for ABCN1, and 1075 °C for ABCN2).

Pellet densities were measured by the displacement of water using the Archimedes method. Field emission scanning electron microscopy (FESEM, Nova NanoSEM230, USA) was used to characterize the surface morphology of polished and thermally etched (at 1030 °C for 30 min) surfaces, with energy dispersive spectroscopy (EDS) performed using an Oxford Instruments EDS system on the same microscope. Grain sizes were measured using the ImageJ software.^[^ ^67^ ^]^ X‐ray powder diffraction (XRD, Cubix diffractometer, PANalytical Cambridge, UK) data were collected using Ni‐filtered Cu‐Kα radiation (λ = 1.5418 Å) on powdered samples at room temperature in flat plate Bragg–Brentano geometry over the 2θ range 5–120° with a step width of 0.03° and an effective count time of 200 s per step. The Rietveld method was used to analyze crystal structures with the GSAS suite of programs.^[^ ^68^ ^]^ Starting models for the Rietveld analysis were based on the structures presented by Farid et al.^[^ ^33^ ^]^ in space group *Pb*2_1_ *m* for pure AN and *Pbcm* for the *x* = 0.006 and *x* = 0.019 compositions (transformed from *P*2_1_ *am* and *Pcam*, respectively, in the original work). Raman spectroscopy was performed on a LabRAM HR800 spectrometer (Horiba JobinYvon, Paris, France). Ag electrodes were used for electrical measurements and applied to pellet faces using Ag‐paste with subsequent decomposition at 550 °C for 30 min. The pellets were poled using a polarization device in a silicone oil bath at room temperature. The temperature and frequency dependence of dielectric properties were measured using Agilent E4980A and 4294A LCR meters, respectively, the former connected to a computer‐controlled furnace. Before measuring energy storage and charge–discharge properties, the samples were polished down to 170–210 µm and coated with electrodes of 2 mm in diameter. The temperature and frequency‐dependent displacement‐electric field (*D–E*) and current–electric field (*I–E*) loops, as well as the cycling stability for these samples, were measured on a ferroelectric measurement system (aixACCT, TF Analyzer 2000/3000, Germany). The charge–discharge properties were measured at different applied electric fields using a commercial charge–discharge system (CFD‐003, Tongguo Technology, Shanghai, China).

## Conflict of Interest

The authors declare no conflict of interest.

## Supporting information



Supporting Information

## Data Availability

The data that support the findings of this study are available from the corresponding author upon reasonable request.
