# Supplementary material for: Phase Transitions in Bi/Ca Modified AgNbO3 Ceramics with Excellent Energy Storage Density and Storage Intensity
Source: Small. 2025 Jun 16;21(32):2500810. doi: 10.1002/smll.202500810 (PMC12366252; doi:10.1002/smll.202500810)
Supplement: Supplementary file 1 — Supporting Information [file SMLL-21-2500810-s001.docx]

Supporting Information

Phase transitions in Bi/Ca modified AgNbO_3_ ceramics with excellent energy storage density and storage intensity

Zhongna Yan, Jia He, Haiyan Chen, Dou Zhang*, Yuan Liu, Hang Luo, Chuanchang Li, Isaac Abrahams*, Haixue Yan*


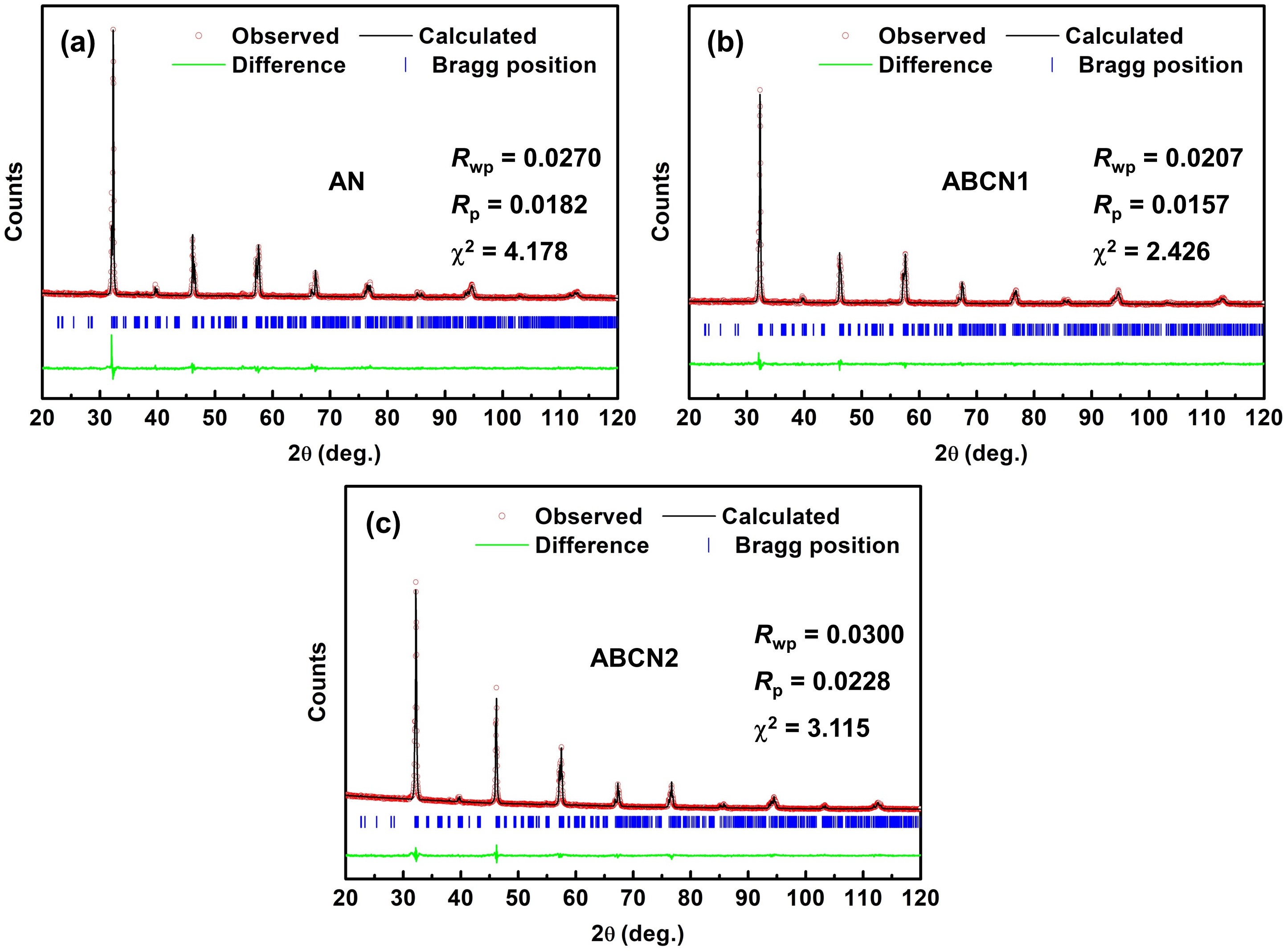


**Fig. S1.** Fitted room temperature diffraction profiles for Ag_(1-5_*_x_*_)_Bi*_x_*Ca*_x_*NbO_3_ ceramic powders using space groups *Pb*2_1_*m* for AN and *Pbcm* for ABCN1 and ABCN2. ^[1]^


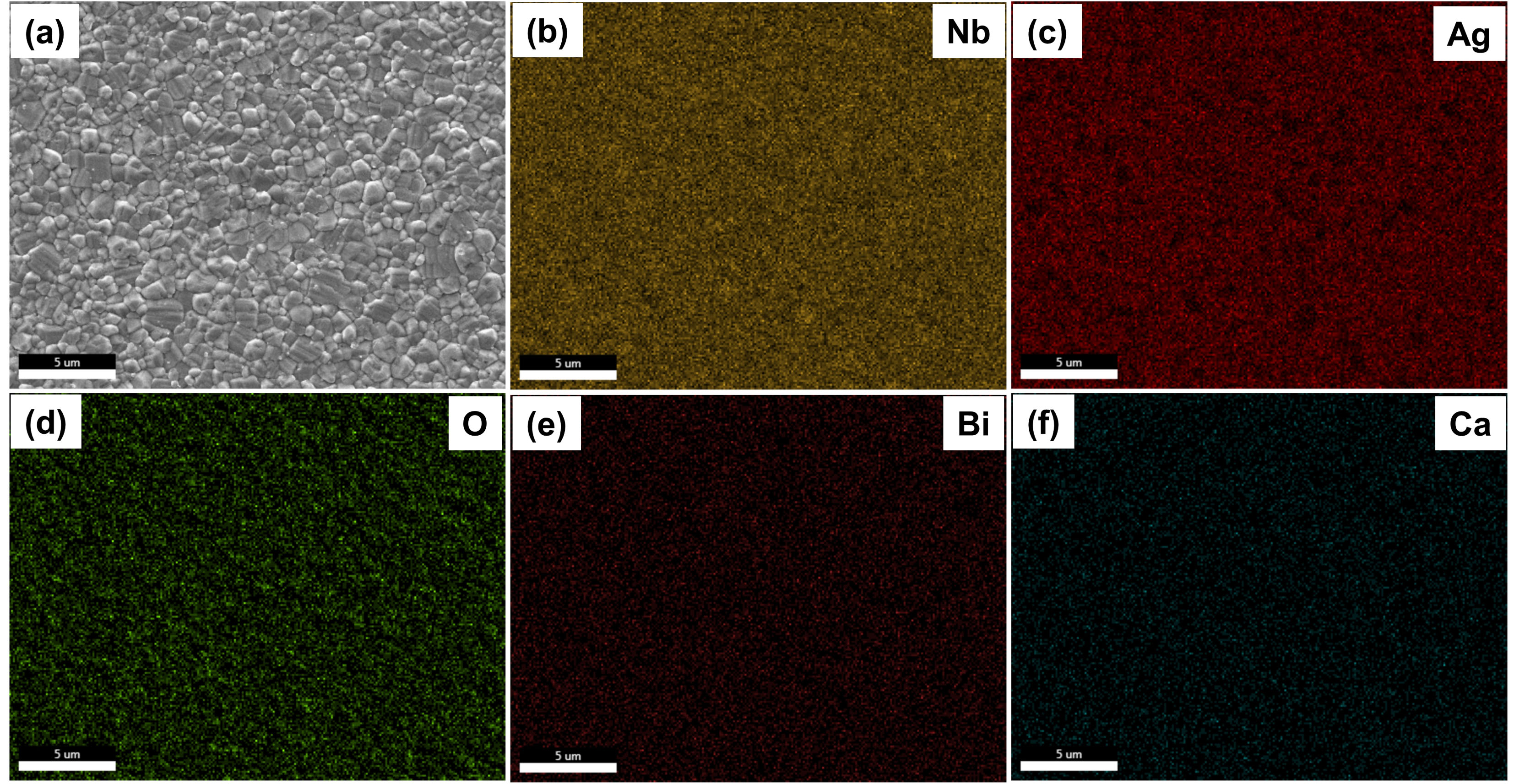


**Fig. S2.** EDS mapping of the ABCN1 ceramic.


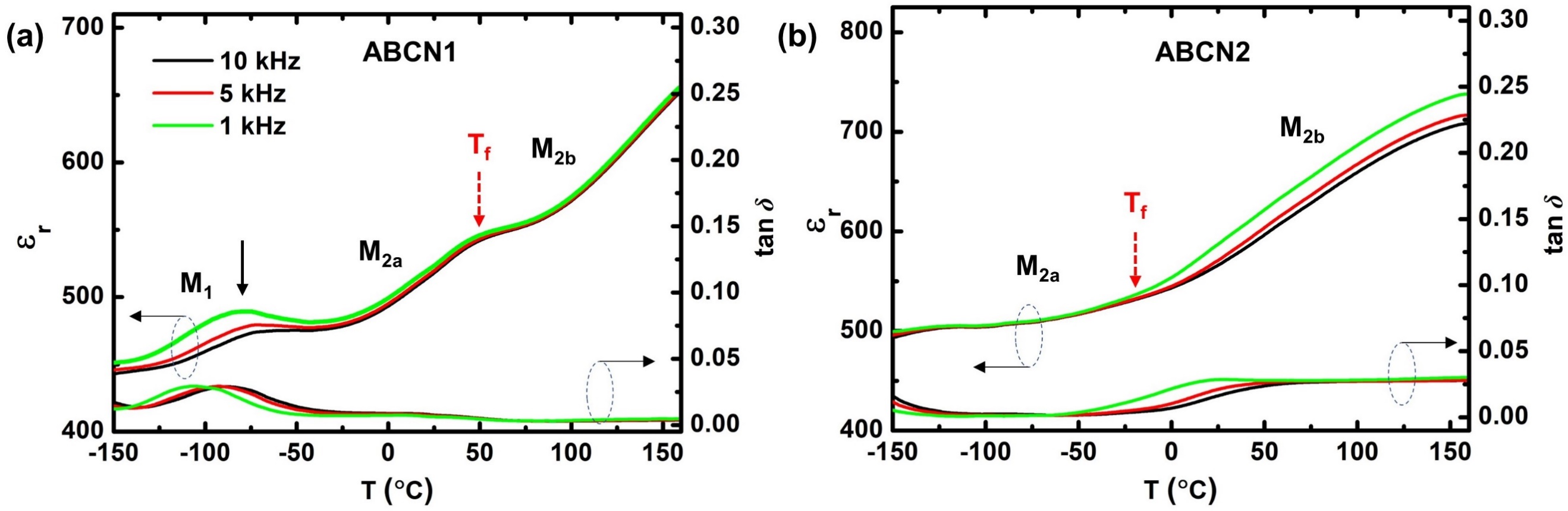


**Fig. S3.** Low temperature dependencies of relative permittivity and loss tangent (tan *δ*) for (a) ABCN1 and (b) ABCN2 ceramics.

Reference

[1] U. Farid, A. S. Gibbs, C. B. J. Kennedy, *Inorg. Chem.* **2020**, 59, 12595.
